# A case study of lean digital transformation through robotic process automation in healthcare

**DOI:** 10.1038/s41598-024-65715-9

**Published:** 2024-06-25

**Authors:** Wei-Lun Huang, Shu-Lang Liao, Hsueh-Ling Huang, You-Xuan Su, Jih-Shuin Jerng, Chien-Yu Lu, Wei-Sho Ho, Jing-Ran Xu

**Affiliations:** 1 Medical Affairs Office, National Taiwan University Hospital, No. 7, Zhongshan S. Rd., Zhongzheng Dist., Taipei, 100225 Taiwan; 2 Department of Industrial Education and Technology, National Changhua University of Education Bao-Shan Campus, No.2, Shi-Da Rd, Changhua, 500208 Taiwan; 3 Department of Health Services Adminstration, China Medical University, No. 100, Sec. 1, Jingmao Rd., Beitun Dist., Taichung, 406040 Taiwan; 4 Department of Ophthalmology, National Taiwan University Hospital, No. 7, Zhongshan S. Rd., Zhongzheng Dist., Taipei, 100225 Taiwan; 5 College of Medicine, National Taiwan University, No. 1, Sec. 1, Ren’ai Rd., Zhongzheng Dist., Taipei, 100233 Taiwan; 6 Center for Quality Management, National Taiwan University Hospital, No. 7, Zhongshan S. Rd., Zhongzheng Dist., Taipei, 100225 Taiwan; 7 Department of Internal Medicine, National Taiwan University Hospital, No. 7, Zhongshan S. Rd., Zhongzheng Dist., Taipei, 100225 Taiwan; 8 NCUE Alumni Association, National Changhua University of Education Jin-De Campus, No. 1, Jinde Rd., Changhua, 500207 Taiwan

**Keywords:** Lean six sigma, DMAIC, RPA, Lean digital transformation, Healthcare improvements, Health care, Health care economics, Health policy, Public health

## Abstract

Under Taiwan's National Health Insurance (NHI) system, it's crucial for all healthcare providers to accurately submit medical expense claims to the National Health Insurance Administration (NHIA) to avoid incorrect deductions. With changes in healthcare policies and adjustments in hospital management strategies, the complexity of claiming rules has resulted in hospitals expending significant manpower and time on the medical expense claims process. Therefore, this study utilizes the Lean Six Sigma DMAIC (Define, Measure, Analyze, Improve, Control) management approach to identify wasteful and non-value-added steps in the process. Simultaneously, it introduces Robotic Process Automation (RPA) tools to replace manual operations. After implementation, the study effectively reduces the process time by 380 min and enhances Process Cycle Efficiency (PCE) from 69.07 to 95.54%. This research validates a real-world case of Lean digital transformation in healthcare institutions. It enables human resources to be allocated to more valuable and creative tasks while assisting hospitals in providing more comprehensive and patient-centric services.

## Introduction

**Figure 1:**
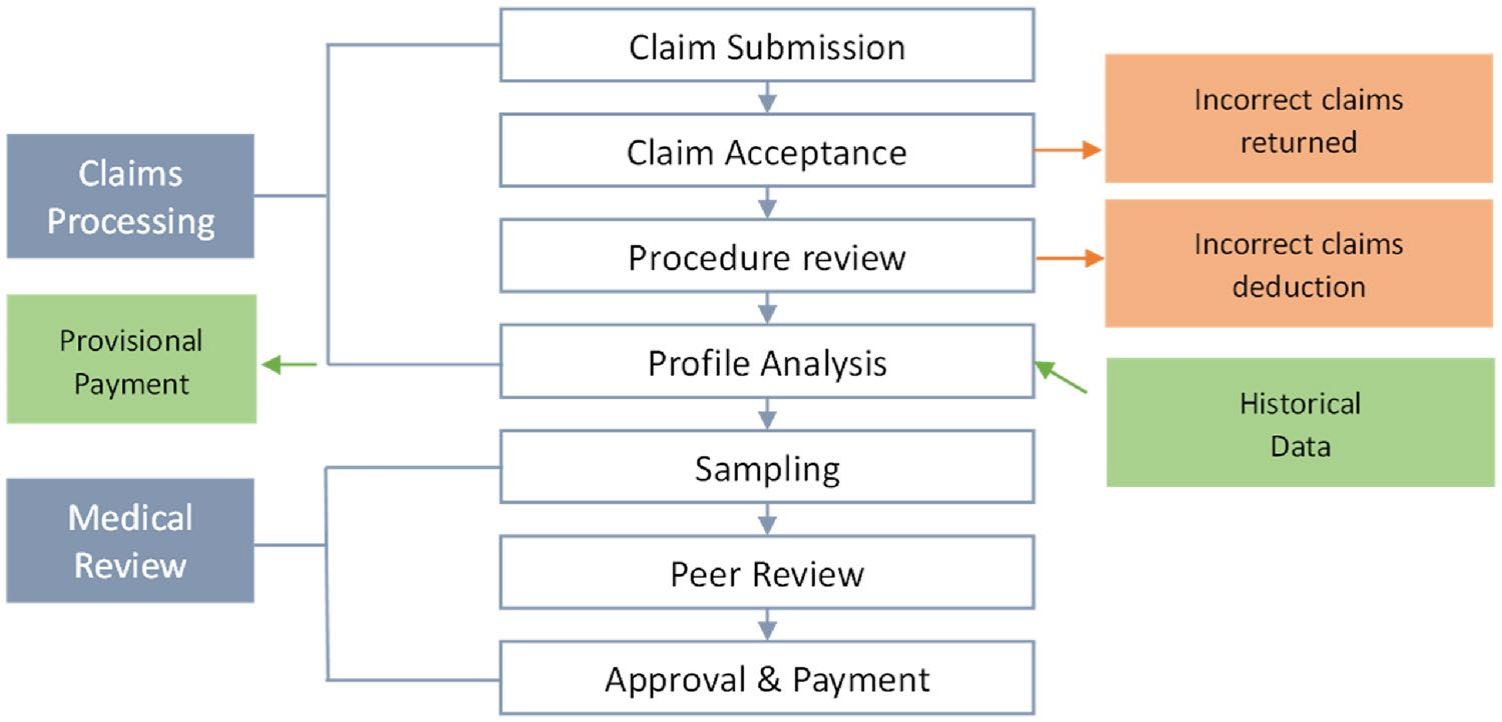
Medical expense claim and review process.

Taiwan's healthcare system operates under the National Health Insurance (NHI), which is a single-payer system. This system ensures that all insurers have fair access to medical services by distributing risks and medical expenses among both healthy and non-healthy individuals. The employed population pays insurance premiums based on their salary income. Veterans and individuals with special identities receive assistance with their insurance and medical expenses^1^. The National Health Insurance (NHI), managed by the National Health Insurance Administration (NHIA), operates with both Retrospective Payment System (RPS) and Prospective Payment System (PPS). Over 93% of healthcare service providers have signed contracts with the NHIA to offer medical services to insured individuals. Among them, the fee-for-service model is the primary payment method for most medical procedures. The "Expert Consultation Meeting on NHI Fee Schedule and Reference List for Medical Services," which includes professionals from the government, healthcare sector, academia, and insurance beneficiaries, collaboratively develops the NHI Fee Schedule. Hospitals claim medical expenses to the NHIA based on the services provided by their medical personnel, including consultations, examinations, procedures, surgeries, and anesthesia, following the provisions in the NHI Fee Schedule^2^. However, depending on the specific medical situation, reimbursement may also be based on case payment, per-diem payment, and Diagnosis Related Groups (DRG)^3^. To prevent unnecessary or inappropriate healthcare services and ensure appropriate reimbursement for necessary and legitimate healthcare providers, the NHIA has established procedures for claims submission, payment verification, and review, as illustrated in Fig. 1. Cases that do not meet eligibility, coverage scope, or payment standards are deducted during verification^4^. Although the NHI reimbursement scheme can effectively utilize medical care resources, it also affects market competition and adds complexity to hospital management^5^.

To ensure that all medical procedures are correctly claimed to the NHIA. National Taiwan University Hospital (NTUH) has established a comprehensive Hospital Information System (HIS), which includes more than 10 subsystems. When a doctor issues an order through the clinic system, the orders are then transmitted to the relevant subsystems for further work. This system effectively manages the recording, pricing, and declaration process for various medical procedures^6^. Under effective management, the claims process can maximize revenue for hospitals while incurring the same medical expenses. Accurate medical claims can also prevent audits and deductions by the NHIA, minimizing financial losses and alleviating patient concerns about medical expenses. With the changes in health policy and hospital strategy, the associated verification procedures have been continuously increasing, leading to a significantly time-consuming and complex operation.

In today's healthcare landscape, hospitals are facing the dual challenge of meeting patients' demands for better care while satisfying health insurers' expectations for lower costs. "Lean" is an effective approach for process improvement and enhancing operational efficiency. It aims to eliminate waste and inconsistencies within production systems. This helps hospitals achieve seemingly conflicting objectives^7,8^. Lean thinking can assist healthcare professionals reduce waiting times and prevent errors, ultimately improving the quality of care and reducing costs. By removing barriers in the process, physicians can focus more on caring for patients^9^. Healthcare professionals and managers have been attempting to use lean tools and techniques to improve the efficiency, clinical outcomes, satisfaction, and safety of both employees and patients, ultimately aiming to enhance financial performance and sustainability^10^.

With the progress of science and technology, Industry 4.0 technology is commonly used as a solution to introduce lean thinking^11^. The adoption of technologies such as Cyber-Physical Systems (CPS), Big Data, Internet of Things (IoT), and Robots does not conflict with the integration of Lean Production System. It increases the flexibility and productivity of enterprises and enables better alignment with future market demands^12^. The technologies of Industry 4.0 can also lead to process improvements in the healthcare industry. More healthcare sectors are embracing Industry 4.0 technologies for digital transformation. The goal is to enhance data digitization, improve interconnectivity between machines and commands, and create more efficient databases. The entire healthcare system is moving towards Healthcare 4.0^13^. It is widely believed in academia and industry that the next wave of transformation will be driven by technology. Therefore, the application of lean thinking to the concept of digital transformation has emerged^14^.

The Industry 4.0 revolution brings many new technologies to industries, enhancing competitiveness through improved quality, reduced costs, and shorter lead times for businesses. However, even with the attractive prospects of digitalization, we should not forget that the principle of lean production is based on the concept of "respect for people"^15^. In the tools of digital transformation, Robotic Process Automation (RPA) can be viewed as a technological tool with human-centric features. It can replace human involvement with software robots in highly repetitive and logical tasks^16^. These software robots can independently execute and schedule tasks quickly, flawlessly, and with traceability, resulting in lower costs compared to traditional automation methods^17^. Numerous studies have demonstrated the effectiveness of RPA technology in enhancing operational efficiency, reducing manpower costs by 20–50%, decreasing workload, automating manual task execution, and improving process cycle efficiency by 30–70%^18^. RPA is considered a leading technology that accelerates digital transformation by connecting manual operations and automated systems^19^. Hospitals manage complex internal and external information, such as medical records, examination data, medical fees, and cross-system data, requiring comprehensive Hospital Information Systems (HIS) to manage operations, finances, and clinical care. Currently, many manual tasks can be completed through human–computer interaction. Introducing RPA could further assist human operators in handling computer tasks, helping hospitals enhance operational efficiency, reduce costs, and avoid human errors^20–22^.

Based on a retrospective study conducted by de Barros et al. regarding the application of Lean tools in the healthcare domain, DMAIC, VSM, SIPOC, Ishikawa Diagram, and 5S emerged as the most frequently utilized Lean tools. The Lean Six Sigma (LSS) methodology has been widely adopted and has demonstrated several positive results, such as increased time for direct patient care, reduced unnecessary procedures, and lower rates of nosocomial infection^23^. Schumacher's research suggests that the digital transformation brought about by the Industry 4.0 technological revolution is intricately linked with and can enhance lean production systems^24^. Furthermore, the combination of LSS with RPA is more powerful and robust compared to using these two methods independently. LSS can optimize processes in terms of quality, speed, efficiency, and resource consumption, while RPA assists in achieving automation and enhancing efficiency^25^. Additionally, the implementation of RPA can eliminate the eighth waste in lean management, known as "the Waste of Talent." This waste occurs when highly skilled, technical, and experienced talent find themselves engaged in repetitive, time-consuming tasks to align with process execution, resulting in talent waste and employee dissatisfaction. RPA allows employees to focus on more value-added tasks^26^. Financial institutions that combine LSS and RPA for digital transformation derive additional value in aspects such as process improvement, structured methodologies, RPA optimization, and resource conservation. They believe that the integration of these two methods prevents inefficient behaviors. LSS accurately identifies processes that need automation, while RPA achieves process optimization^27^.

In recent years, there has been increasing research attention on the impact and correlation between Lean and Industry 4.0, but limited research has presented practical methods and cases that combine the two^28^. Additionally, many businesses may fail in their digital transformation efforts because they excessively focus on adopting new technologies while overlooking the motivation for "process-centric" improvement^15^. This study combined the application of lean six sigma (LSS) management methods with Robotic Process Automation (RPA) technology tools to analyze the National Taiwan University Hospital's medical expense claims process, identify waste and key improvement steps in the process, and then automate and optimize the workflow to improve operational efficiency.

## Results

**Figure 2:**
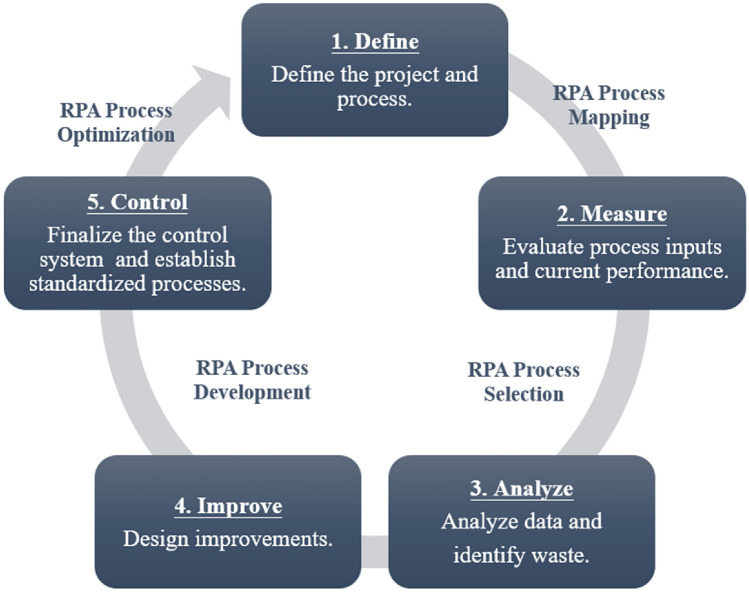
DMAIC five stages.

In this research, we applied the Lean Six Sigma DMAIC framework in conjunction with RPA for process improvement and digital transformation. The DMAIC process consists of five phases: Define, Measure, Analyze, Improve, and Control. The implementation of RPA completes its mapping, selection, development, and optimization steps within the framework of the DMAIC process, as illustrated in Fig. 2.

### Define phase

In the definition phase, it is essential to clarify the project scope, identify the issues to be addressed, and determine the processes to be improved to establish project objectives. This study prepared a project charter, encompassing the project name, voice of customer, objectives, expected benefits, and other information, to ensure that project team members are aligned towards common goals, as shown in Table 1.

**Table 1:** Project charter.

Project charter	
Project name	Improving efficiency in medical expense claims process project
Project champion	Medical affairs office director
Project owner	Medical affairs office
Team members	Medical affairs office supervisor Quality management center supervisor 12 staff in the medical affairs office
Problem statement	The medical expense claims process is cumbersome, consuming significant manpower and time resources
Aim of the project	To reduce or eliminate inefficiencies in medical expense claims process by applying DMAIC and RPA
Voice of customer	Internal customers (employees) perceive the workflow as cumbersome with numerous manual tasks External customers (patients) indicate that the inability to promptly provide accurate medical expense reimbursements leads to issues in repeated visits to hospitals for resolution
Expected benefits	Enhanced process efficiency and accuracy in claims submission, resulting in increased satisfaction among both patients and employees

**Figure 3:**
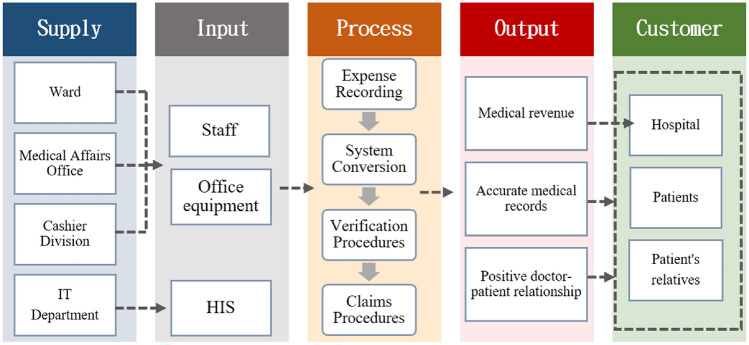
SIPOC model.

This study utilizes the SIPOC model to define the scope of the process. In this medical expense claims process, resource requirements include personnel from various departments, medical information systems, and office equipment. Suppliers include the ward, Medical Affairs Office, Cashier Division, and IT department. Core processes involve expense recording, system conversion, verification procedures, and claims procedures. Output variables include medical revenue, accurate medical records, and positive doctor-patient relationships. Hospitals are the ultimate customers for medical revenue. Accurate medical records and positive doctor-patient relationships benefit hospitals, patients, and their families, as illustrated in Fig. 3.

The ward unit is the origin of all medical activities, and medical expenses are generated by the ward clerks after physicians issue medical orders or treatments. This process is also prone to accounting errors, especially during busy healthcare procedures. NTUH has allocated 11 employees to conduct daily checks on patient information and medical expense records according to reports generated by the IT department. These employees need to verify whether patients are billed according to their respective statuses and ensure that the medical orders comply with the NHI Fee Schedule. In case of anomalies, they must reach out to patients for missing information or inform ward clerks to rectify erroneous records. Upon discharge from the hospital, the Medical Affairs Office sends the medical bills to patients before discharge from the hospital and finalizes the electronic claims procedures.

During the definition phase, this study identified that verifying of medical expenses is the step within the core process that requires the highest manpower and time investment. Therefore, this study thoroughly elucidates and documents the sequential process steps involved in the verification procedures, while simultaneously conducting process mapping for RPA implementation to facilitate subsequent phases.

### Measure phase

The process of daily verification of medical expenses includes the following steps: "Employees receive reports via email → Open each report individually → Filter the assigned wards → Verify any abnormal issues → Handle abnormal incidents". Employees need to review a total of 28 reports (as shown in Table 2), including 9 reports for patient status verification, 8 reports for claim data verification, 8 reports for ward verification, and 3 reports for expense verification.

**Table 2:** Reports list.

Report type	Report name
Patient status verification	1. Major illness abnormalities 2. Self-pay patient status abnormalities 3. Hospitalized patients with major illness but no relevant diagnosis 4. Differences in emergency to inpatient admission status 5. Hospital admissions without reading NHI IC card 6. Discharges without reading NHI IC card 7. Abnormal health insurance patient status 8. Health insurance patient status without co-payment codes 9. Newborns using mother's health insurance status after 60 days of birth
Claim data verification	1. Special medical orders during hospitalization 2. Usage of special exemption from co-payment status during hospitalization 3. Hospitalized patients with infections notifications 4. Changes in medical procedures during hospitalization 5. Rehospitalization within 14 days of discharge 6. Unexecuted medical orders within 7 days after discharge 7. CVA (cerebrovascular accident) status the previous day 8. Usage of referral patient status
Ward verification	1. Transferred patients within the hospital 2. Hospitalized patients entering and discharging isolation wards 3. Hospitalized patients entering and discharging palliative care wards 4. Hospitalized patients entering and discharging psychiatric isolation wards 5. Hospitalized patients entering and discharging obstetrics wards 6. Hospitalized patients entering and discharging baby rooms 7. Hospital discharges of patients under 12 years old 8. Patients remaining in discharge preparation for over 5 days
Expense verification	1. Unsettled medical expenses on the day of discharge 2. Patients with unsettled payments readmitted 3. Usage of pediatric medical expense subsidy

NTUH has a total of 88 ward divisions, managed by 11 employees. Due to differences in the characteristics of the wards assigned to each employee, the time required for verification tasks also varies. During the three-month observation period, this study calculated the time-related data of employee operation systems through the backend of the information system. The results indicate that the average individual process time is 110.9 min, with a total process time of 1220 min. Among the different check categories, the highest total check time is 281 min for claim data, followed by 252 min for patient status checks and 240 min for ward checks. We also observed that the total cumulative operation time amounts to a remarkable 377 min. The average time consumed for each step is shown in Table 3.

**Table 3:** Process time (unit: minutes).

Employee	Patient status verification	Claim data verification	Ward verification	Expense verification	Operation time	Individual process time
A	18	16	24	4	30	92
B	22	26	21	6	36	111
C	20	21	19	5	31	96
D	21	24	22	5	32	104
E	21	26	21	6	38	112
F	28	32	32	12	40	144
G	27	27	26	7	37	124
H	21	23	18	8	34	104
I	26	28	21	7	31	113
J	25	31	19	6	33	114
K	23	27	17	4	35	106
Total time	252	281	240	70	377	1220
Average time	22.9	25.5	21.8	6.4	34.3	110.9

### Analyze phase

**Figure 4:**
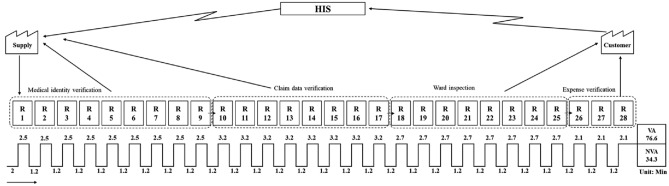
Value stream mapping.

Based on the data observed during the measurement phase, a Value Stream Map (as depicted in Fig. 4) was created. Due to the intricate nature of human–computer interactions, the process was visualized in a clear and understandable flowchart. Under the condition of consistent fields and characteristics among reports of the same functional category, this study calculates the average verification time of each report by using the process time for each step as recorded in Table 2, along with the number of reports for that step. Additionally, it determines the average time for each preceding action by considering the operation action time and the frequency of the same action. The process examined in this study does not incorporate inventory activities or waiting processes for report handling. Therefore, such indications were not depicted in the VSM diagram.

In Lean thinking, value and waste are relative concepts typically identified from the customer's perspective. Activities or products that meet customer needs and expectations are considered valuable, while those that do not add value are viewed as waste. We define steps such as patient identity status, claim data verification, ward verification, and expense verification as value-added steps for both patients and hospitals. From the customer's perspective, patients should pay medical expenses based on accurate medical records, and hospitals should receive appropriate revenue after processing the claims. The purpose of reviewing these reports is to reduce claim errors and promptly handle any exceptional events. However, we have also observed that actions such as receiving emails, opening reports, and filtering cells within the process, while not directly adding value to patients or hospitals, are essential tasks required to carry out the work. These steps, which can be automated, should not be performed manually daily. They are suitable for RPA implementation to handle tasks involving various interfaces, software switches, and operations. Additionally, during our process review, we have observed that opening reports and completing filtering steps are often unnecessary when there are no exceptional events in the assigned areas. This is another process waste that should be eliminated.

This study calculated the value-added time of the verification process to be 76.6 min, with a total process time of 110.9 min. The results indicate the Process Cycle Efficiency (PCE) of 69.07% (76.6 min/110.9 min). Therefore, the aim of this study is to develop RPA for critical steps, eliminate wasteful steprs in the process, or reduce the time spent on non-value-added steps.

### Improve phase

Based on the aforementioned analysis results, this study introduces RPA technology tools to replace manual, daily, repetitive non-value-added tasks and completes the development process following the steps outlined below.

### Preparatory work

Re-inventory and review all reports, and ask the IT department to help standardize specific fields in all reports, and reorganize the logic conditions for capturing abnormal data in each report. Remove outdated reports that no longer meet current needs, simplify and eliminate unnecessary processes to facilitate the subsequent establishment of RPA for data capture.

### Develop RPA

**Figure 5:**
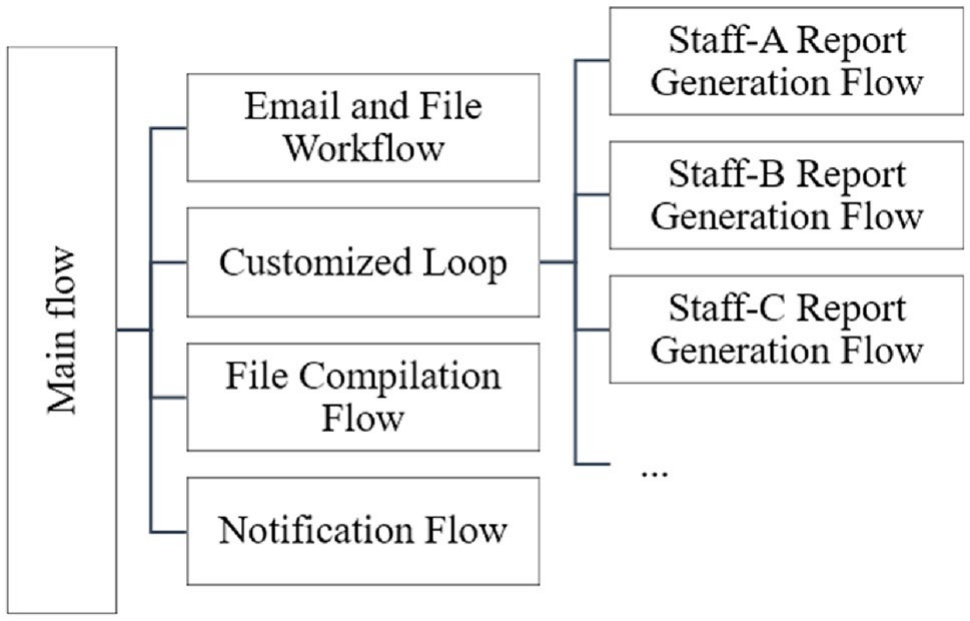
System process architecture.

This study used Microsoft Power Automate version 2.38.176.23294 to develop the RPA solution. The RPA solution involved utilizing various functionalities, such as email extraction and sending, file processing, Excel operations, list and variable configuration, to achieve robot automation configuration. The design of process automation involved integrating main processes with sub-processes to facilitate future maintenance and adjustments. The architecture of the automated process design is illustrated in Fig. 5.

**Figure 6:**
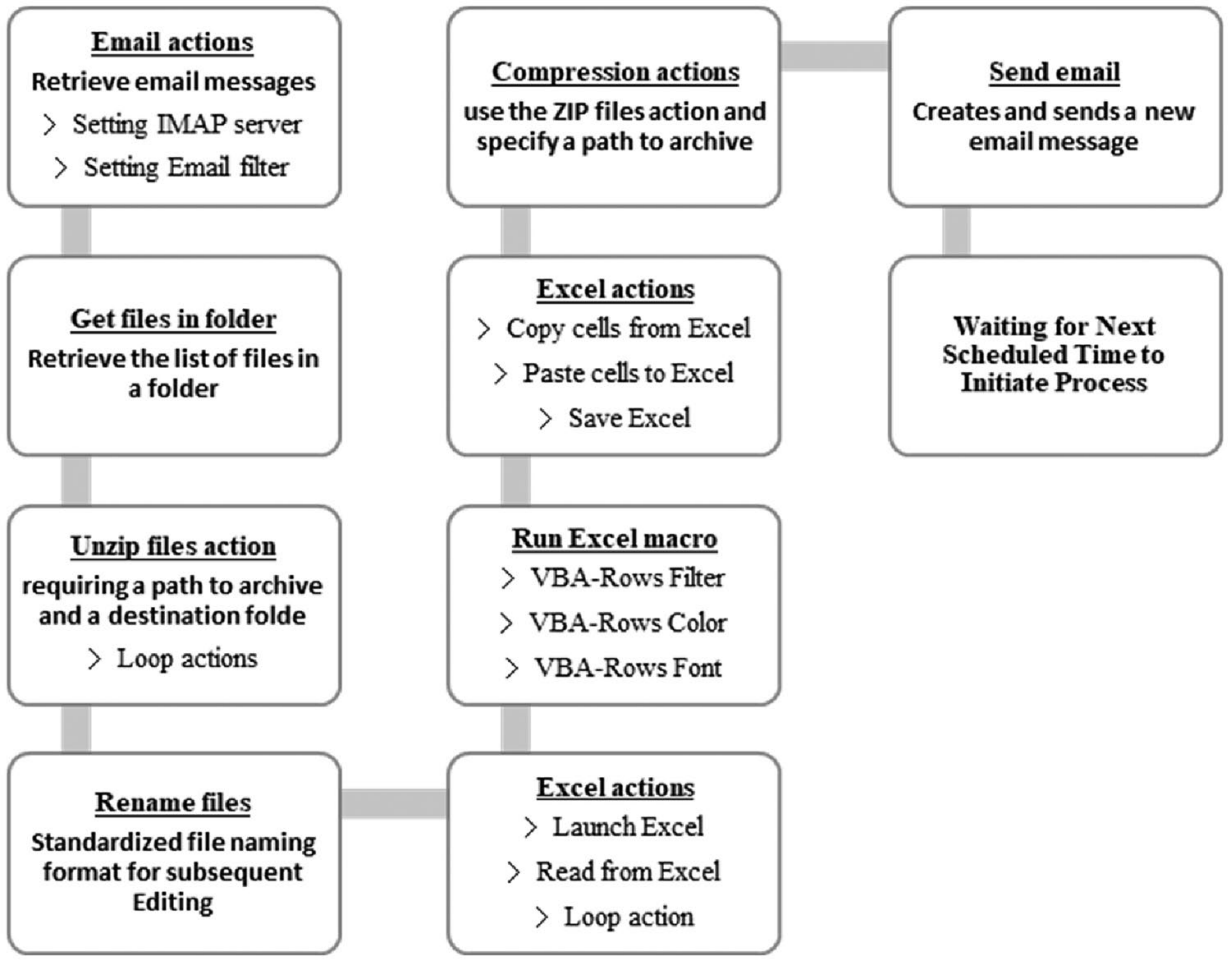
RPA workflow.

This process is scheduled to automatically retrieve specific emails daily to obtain reports generated by the IT Department. It then proceeds with actions such as file storage and decompression. After consolidating 28 reports, the program enters a loop to customize individual reports for each employee. Multiple Excel VBA programs automatically divide the reports, which cover all hospital wards, into 11 reports corresponding to each employee's assigned ward sections. We evaluated the importance of reports by analyzing on the frequency of abnormal event occurrences, their impact on clinical care quality, hospital finances, and other related aspects. The prioritization of report importance was determined through multiple meetings and weighted scoring. A unified report layout, font, and color design were established to achieve visual presentation. Following the file editing process, the reports are automatically sent to the respective employee's mailbox. This system enables employees to open only one report per day to initiate data verification and case processing, replacing the previously time-consuming and repetitive task of individually opening and filtering multiple reports. This eliminates wasteful steps and reduces non-value-added time in the process. The RPA workflow is illustrated in Fig. 6.

### Control phase

During the development of RPA, all execution steps were fully recorded, and records were kept throughout program execution. In the future, when there is a need to add or delete reports, users can adjust the automation process themselves and put it online immediately. Therefore, RPA not only brings digital transformation to the process but also facilitates process maintenance and the transfer of experience.

### Implementation outcomes

After implementing the RPA improvement solution in the medical expense claims process, this study re-measured the time spent by employees in each step of the verification process, as shown in Table 4. The results indicate that the total process time has been reduced from 1220 min to 840 min, resulting in a reduction of 380 min. Among them, there was a reduction of 340 min in non-value-added processes. Additionally, due to consolidating reports into a single document with adjusted importance order and visual layout, there was a 40-min reduction in the value-added process during the actual verification step.

**Table 4:** The improved process time (unit: minutes).

Employee	Patient status verification	Claim data verification	Ward verification	Expense verification	Operation time	Individual process time
A	18	16	21	4	2	61
B	21	25	19	5	3	73
C	20	20	18	4	3	65
D	20	22	22	5	4	73
E	19	24	19	5	4	71
F	26	30	31	9	6	102
G	27	26	25	6	5	89
H	21	23	18	7	3	72
I	25	27	21	7	2	82
J	25	30	19	6	2	82
K	22	27	15	3	3	70
Total time	244	270	228	61	37	840
Average time	22.2	24.5	20.7	5.5	3.4	76.4

The average verification time per person decreased from 110.9 to 76.4 min, with a reduction of 30.9 min in the time spent on operational tasks. Overall, the average verification time per person decreased by 3.3 min. Therefore, the calculated process cycle efficiency improved from 69.07 to 95.54%, representing a growth of 26.47%, as shown in Table 5.

**Table 5:** Comparison of process time before and after (unit: minutes).

Item	Before (A)	After (B)	Difference (B − A)
Patient status verification	252	244	−8
Claim data verification	281	270	−11
Ward verification	240	228	−12
Expense verification	70	61	−9
Operation time	377	37	−340
Total process time	1220	840	−380
Average time	110.9	76.4	−34.5
Value-add time	76.6	73	−3.3
Non-value-add time	34.3	3.4	−30.9
Process cycle efficiency (%)	69.07	95.54	26.47

## Discussion

Utilizing information technology tools for business process automation ensures compliance with legal and ethical standards and enhances internal control. Research also indicates that leveraging technology to maintain electronic healthcare records improves both the performance of healthcare services and cost efficiency^29^. Combining the methods of Lean Healthcare can establish a prioritization of digital transformation, helping to avoid the adoption of technologies less likely to improve the value stream, thereby preventing the waste of human resources and costs^30^. A study successfully integrated Healthcare 4.0 with lean methodologies in a case study within the sterilization unit of a large public university hospital. The research involved mapping current and future value stream maps, collecting feedback and knowledge-related data on the applications used, and ultimately evaluating the importance of each application to prioritize value stream integration. This approach effectively leveraged lean tools to drive the digital transformation of healthcare services^31^. During the discussion phase of this study, we also evaluated the possibility of modifying and configuring the method for producing reports within the information system. However, it was discovered that not only impacted the efficiency of database operations but also required a longer development time, making subsequent adjustments and maintenance more challenging. Therefore, this case introduced RPA as a process improvement solution within the DMAIC framework. Unlike the classical "inside-out" approach of information technology improvement, RPA involves automating processes "outside-in" by replacing human effort, providing a cost-effective solution without altering the original information system^32^. A systematic review investigated the impact of dual interventions involving Industry 4.0 simulation modeling techniques and lean methodologies on healthcare services. The findings indicated that most studies focused on patient flow metrics such as hospital stay duration, waiting times, and turnaround times, with limited evidence on outcomes related to patient health, employee well-being, and resource utilization. Therefore, it is recommended to enhance staff participation in the lean digital transformation process^33^. RPA software utilizes a graphical interface, eliminating the need for advanced programming language skills to develop robots, making it an effective tool for enhancing employee participation in lean improvement processes. Compared to the traditional organizational reliance on IT departments when introducing information technology, frontline workers (process owners) can independently develop relevant robots during RPA implementation. The automation process completed by the users themselves can also better meet user needs^34^.

In digital transformation, adjustments to existing processes or the purchase of additional software and hardware equipment are often necessary. However, the purpose of using technology tools should be to serve human beings and work processes, rather than creating new processes and obstacles that conflict with the principles of lean thinking and eliminating waste^14^. Digitizing and automating a process that is full of waste will only result in the automation of waste. Therefore, applying lean thinking to eliminate waste, identify repeatable processes that can be standardized, and manage complex processes that need to be controlled can help facilitate digital transformation^35^. In the lean digital transformation program, it is also important to redefine the definition of waste. It encompasses not only physical waste but also digital waste, such as storing and retrieving unnecessary data that consumes database resources. Additionally, computer capabilities are still limited and cannot solve all optimization problems. Managers cannot rely solely on machines and people remain the core role in the process^36^. Therefore, the lean digital transformation should focus on the goals we want to achieve rather than the technically feasible goals. By using lean thinking to identify value and waste, we can then evaluate which technology is best suited to achieving our goals, allowing improvement actions to complement the supporting technology for maximum benefit.

This study utilized Lean Six Sigma tools to identify key improvement steps in the process and implemented RPA as a solution. By replacing manual tasks with software robots, the study reduced non-value-added time and steps associated with repetitive operations, as well as eliminated unnecessary steps and waste in the process. After the improvement, the total process time was significantly reduced by 380 min. Furthermore, a one-page report format eliminated the previously wasteful steps in the process. The overall process cycle efficiency increased from 69.07 to 95.54%. This efficiency improvement not only enhances external customer satisfaction but also increases internal customer satisfaction as internal processes become more efficient and waste is reduced.

The integration of LSS with RPA represents a pioneering approach to digital transformation within hospital settings. By leveraging lean management principles alongside advanced artificial intelligence tools, this innovative strategy has resulted in a significant reduction in operating costs. Moreover, by prioritizing 'people-centric' improvement solutions over purely 'technology-centric' approaches, the initiative has successfully offloaded repetitive tasks to robots, thereby enabling healthcare professionals to focus on delivering warm and personalized services. This study not only offers valuable insights for future research but also extends support to industries embarking on Lean Management and Digital Transformation initiatives, demonstrating the potential for impactful and sustainable change.

## Methods

This study employed the case report research method to directly observe operational challenges in hospitals^37,38^. Implemented at National Taiwan University Hospital (NTUH), a prominent teaching hospital in Taipei, Taiwan, with over 2600 beds and 6700 employees, this research delved into the intricate dynamics of hospital operations. NTUH, committed to delivering high-standard healthcare services, continuously drives internal quality improvement initiatives, providing an ideal setting for this investigation.

This study implemented Lean Six Sigma and integrated Robotic Process Automation (RPA) to drive the project. Lean Six Sigma, a combination of lean thinking and Six Sigma methods, focuses on process improvement through waste elimination and reduction of errors and variations using statistical and management tools. The DMAIC cycle (Define, Measure, Analyze, Improve, Control) was primarily adopted as the roadmap for problem-solving^39^. However, there is currently no universally adopted single framework or model for RPA implementation. Different enterprises choose or develop RPA implementation frameworks according to their needs and conditions. Therefore, after searching relevant literature and evaluating our business characteristics, this study summarized four steps: process mapping, process selection, process development, and process optimization, integrating them with the five phases of DMAIC for implementation^40,41^.

In the Define phase, it is essential to clearly define the project scope. Commonly used tools include Gantt charts, SIPOC diagrams, Voice of the Customer (VOC) analysis, and Affinity Diagrams to formulate project charters outlining the problems and objectives. The SIPOC model is utilized to define the scope of processes. Through the five dimensions of Supply, Input, Process, Output, and Customer, it clarifies the stakeholders and elements involved in the overall process, effectively preventing scope creep^42^. This model helps management broaden their perspective and identify the essence of processes. During the Define phase of this study, project charters were formulated based on tools such as the SIPOC diagram and Voice of the Customer analysis.

The objective of the Measure phase is to quantify the current performance of the process and collect relevant data to establish a baseline for comparison. Process maps, scatter plots, and descriptive statistics are used to display the current state. To understand the actual processes, human resources, and time consumption involved in medical expense claims processes, this research employed a field survey approach. During the three-month observation phase, employee activities such as file downloading, opening, closing, and other operations were recorded and calculated through backend information system logs. The verification time of reports is calculated as the difference between the time of opening the report, completing the screening actions, and closing the report. The operation time includes the time spent on actions such as receiving emails, decompressing files, saving files, opening and closing files, and filtering ward, with the time difference between the start and end of each action calculated. The average time consumed for each step is shown in Table 2. This stage also serves as the primary step in RPA implementation—“process mapping.” It involves detailing the current execution process, including system operations, actions, and sequence. Additionally, it lists the variables such as system software and files used.

During the Analysis and Improvement phases, it is essential to identify problems from the measured results and propose appropriate solutions to reduce adverse time and manpower costs. Tools such as Ishikawa diagrams, 5-Why analysis, Value Stream Mapping (VSM), and regression analysis are commonly used to clarify the root causes of issues. Subsequently, problems are addressed through auxiliary methods such as Design of Experiments (DoE) and Failure Mode and Effects Analysis (FMEA)^43^. This study primarily employed Value Stream Mapping (VSM) to visualize the cycle time and information flow across various stages of the process.This aided in identifying of value-added and non-value-added activities, as well as pinpointing key steps for process improvement^44^. Additionally, theProcess Cycle Efficiency (PCE) method proposed by George was utilized to measure process efficiency and identify waste within the process by comparing the time spent on value-added activities to the total lead time^45^. PCE is calculated as (Value Added time)/(total lead time). This stage corresponds to th "process selection" and "process development" steps in the RPA implementation process. By analyzing the time and value contribution of each step during the analysis phase, suitable steps for automation were evaluated to replace manual intervention with robots. Appropriate RPA automation features were selected to develop RPA processes for critical process improvement.

In the Control phase, tools such as Statistical Process Control (SPC), 5S, standardization, and visualization are applied to ensure that the improvement results do not deteriorate. With the implementation of RPA, the development outcomes, recorded as the complete process steps, can be utilized to establish standardized processes. After implementation, records of RPA execution and anomaly notifications are utilized to ensure the successful operation of automation programs, thereby ensuring the sustainability of improvement outcomes. This stage represents the final step of RPA implementation—“process optimization.” Following the overall DMAIC framework implementation with RPA, if issues such as insufficient efficiency improvement or multiple system anomalies arise, the next DMAIC cycle should be initiated to clarify the problems and adjust the automated processes, ensuring continuous improvement of the effectiveness of the enhancements.

### Study limitations

Since this study was conducted in a specific healthcare institution in Taiwan, the results may not be generalizable to other countries or regions, nor can they represent the situation in all hospitals or the entire healthcare system. Although RPA tools demonstrated significant effectiveness in this study, their adaptability and efficacy may vary across different hospitals or processes. This study primarily focused on reducing process time and enhancing cycle efficiency, lacking an assessment of long-term impacts such as employee morale and patient satisfaction. These aspects need to be addressed in future research.

## Data Availability

The datasets used and analyzed during the current study are available from the corresponding author upon reasonable request.

## References

[1] Hsiao WC, Cheng SH, Yip W (2019). What can be achieved with a single-payer NHI system: The case of Taiwan. Soc. Sci. Med..

[2] Yip WC, Lee YC, Tsai SL, Chen B (2019). Managing health expenditure inflation under a single-payer system: Taiwan's National Health Insurance. Soc. Sci. Med..

[3] Wu TY, Majeed A, Kuo KN (2010). An overview of the healthcare system in Taiwan. Lond. J. Primary Care.

[4] Lee, P. C., Wang, J.T. H., Chen, T. Y., & Peng, C. H. *Digital Health Care in Taiwan—Innovations of National Health Insurance*. 1st edn. 10.1007/978-3-031-05160-9 (Springer, 2022).

[5] Chang H, Chang WJ, Das S, Li SH (2004). Health care regulation and the operating efficiency of hospitals: Evidence from Taiwan. J. Acc. Public Policy.

[6] Yang, T. H., Cheng, P. H., Yang, C. H., Lai, F., Chen, C. L., Lee, H. H., & Sun, Y. S. A scalable multi-tier architecture for the National Taiwan University hospital information system based on HL7 standard. In *19th IEEE Symposium on Computer-Based Medical Systems 2006 (CBMS'06)*. 99–104. 10.1109/CBMS.2006.27 (IEEE, 2006).

[7] Jasti NVK, Kodali R (2015). Lean production: Literature review and trends. Int. J. Prod. Res..

[8] Yaduvanshi D, Sharma A (2017). Lean six sigma in health operations: Challenges and opportunities—‘Nirvana for operational efficiency in hospitals in a resource limited settings’. J. Health Manag..

[9] Graban, M. *Lean Hospitals: Improving Quality, Patient Safety, and Employee Engagement*. 3rd ed. 10.4324/9781315380827 (Productivity Press, 2016).

[10] D’Andreamatteo A, Ianni L, Lega F, Sargiacomo M (2015). Lean in healthcare: A comprehensive review. Health Policy.

[11] Mittal S, Khan MA, Romero D, Wuest T (2019). Smart manufacturing: Characteristics, technologies and enabling factors. Proc. Inst. Mech. Eng. Part B J. Eng. Manuf..

[12] Silvestri, L., Gallo, T., & Silvestri, C. Which tools are needed to implement lean production in an industry 4.0 environment? A literature review. *Proc. Comput. Sci.* **200**, 1766–1777 10.1016/j.procs.2022.01.377 (2022).

[13] da Silveira, F., Neto, I. R., Machado, F. M., da Silva, M. P., & Amaral, F. G. Analysis of industry 4.0 technologies applied to the health sector: Systematic literature review. *Occup. Environ. Saf. Health*10.1007/978-3-030-14730-3_73 (2019).

[14] Ashrafian, A. *et al*. Sketching the landscape for lean digital transformation. In *Advances in Production Management Systems. Production Management for the Factory of the Future. APMS 2019. IFIP Advances in Information and Communication Technology* (Ameri, F., Stecke, K., von Cieminski, G., Kiritsis, D. eds.). Vol. 566. 10.1007/978-3-030-30000-5_4 (Springer, 2019).

[15] Romero, D., Flores, M., Herrera, M., & Resendez, H. Five management pillars for digital transformation integrating the lean thinking philosophy. In *2019 IEEE International Conference on Engineering, Technology and Innovation* (*ICE/ITMC*). 1–8 10.1109/ICE.2019.8792650 (IEEE, 2019).

[16] Ivančić, L., Suša Vugec, D., Bosilj Vukšić, V. Robotic process automation: Systematic literature review. In *Business Process Management: Blockchain and Central and Eastern Europe Forum. BPM 2019. Lecture Notes in Business Information Processing* (Di Ciccio, C. *et al*.). Vol. 361. 10.1007/978-3-030-30429-4_19 (Springer, 2019).

[17] Hofmann P, Samp C, Urbach N (2020). Robotic process automation. Electron. Mark..

[18] Syed, R., Suriadi, S., Adams, M., Bandara, W., Leemans, S. J., Ouyang, C., & Reijers, H. A. Robotic process automation: Contemporary themes and challenges. *Comput. Ind*. **115**, 103162 10.1016/j.compind.2019.103162 (2020).

[19] Siderska J (2020). Robotic process automation—A driver of digital transformation. Eng. Manag. Prod. Serv..

[20] Mohapatra S (2015). Using integrated information system for patient benefits: A case study in India. Int. J. Healthc. Manag..

[21] Mohapatra S (2021). Human and computer interaction in information system design for managing business. IseB.

[22] Jain, R., & Bhatnagar, R. Robotic process automation in healthcare—A review. *Int. Robot. Autom. J*. **5**(1), 12–14 10.15406/iratj.2019.05.00164 (2018).

[23] de Barros, L.B., Bassi, L.d.C., Caldas, L.P., Sarantopoulos, A., Zeferino, E.B.B., Minatogawa, V. & Gasparino, R.C. Lean healthcare tools for processes evaluation: An integrative review. *Int. J. Environ. Res. Public Health*. **18**, 7389 10.3390/ijerph18147389 (2021). 10.3390/ijerph18147389 PMC8304063 34299840

[24] Schumacher S, Bildstein A, Bauernhansl T (2020). The impact of the digital transformation on lean production systems. Procedia CIRP.

[25] Maes, J., & Schijns, J. *Lean Robotics. Lean & RPA, de Winnende Combinatie*. https://www.managementboek.nl/boek/9789492790255/lean-robotics-lean-and-rpa-de-winnende-combinatie-john-maes (2020).

[26] Gradim, B., & Teixeira, L. Robotic process automation as an enabler of industry 4.0 to eliminate the eighth waste: A study on better usage of human talent. *Proc. Compu. Sci*. **204**, 643–651 10.1016/j.procs.2022.08.078 (2022).

[27] Schnellmann M, Block A, Gellrich M (2023). Mehrwerte und herausforderungen der kombinierten nutzung von lean six sigma und robotic process automation bei finanzinstituten. HMD..

[28] Taghavi, V., & Beauregard, Y. The relationship between lean and industry 4.0: Literature review. In *Proceedings of the 5th North American Conference on Industrial Engineering and Operations Management*, Detroit, MI, USA. 10–14. http://www.ieomsociety.org/detroit2020/papers/189.pdf (2020).

[29] Mohapatra S, Murarka S (2016). Improving patient care in hospital in India by monitoring influential parameters. Int. J. Healthc. Manag..

[30] Tortorella G, van Dun DH, de Almeida AG (2020). Leadership behaviors during lean healthcare implementation: A review and longitudinal study. J. Manuf. Technol. Manag..

[31] Tortorella GL, Fogliatto FS, Tlapa Mendoza D, Pepper M, Capurro D (2023). Digital transformation of health services: A value stream-oriented approach. Int. J. Prod. Res..

[32] Van der Aalst WM, Bichler M, Heinzl A (2018). Robotic process automation. Bus. Inf. Syst. Eng..

[33] Tlapa D, Franco-Alucano I, Limon-Romero J, Baez-Lopez Y, Tortorella G (2022). Lean, six sigma, and simulation: Evidence from healthcare interventions. Sustainability.

[34] Kokina J, Blanchette S (2019). Early evidence of digital labor in accounting: Innovation with robotic process automation. Int. J. Acc. Inf. Syst..

[35] Boute RN, Gijsbrechts J, Van Mieghem JA (2022). Digital lean operations: Smart automation and artificial intelligence in financial services. Innov. Technol. Interface Finance Oper..

[36] Romero, D., Gaiardelli, P., Thürer, M., Powell, D. & Wuest, T. Cyber-physical waste identification and elimination strategies in the digital lean manufacturing world. In *Advances in Production Management Systems. Production Management for the Factory of the Future. APMS 2019. IFIP Advances in Information and Communication Technology* (Ameri, F., Stecke, K., von Cieminski, G., Kiritsis, D. eds.). Vol. 566. 10.1007/978-3-030-30000-5_5 (Springer, 2019).

[37] Avery A, Cresswell K, Crowe S, Huby G, Robertson A, Sheikh A (2011). The case study approach. BMC Med. Res. Methodol..

[38] Meredith J (1998). Building operations management theory through case and field research. J. Oper. Manag..

[39] Kuei C (2004). Leading six sigma—A step-by-step guide based on experience with GE and other six sigma companies. Int. J. Qual. Reliab. Manag..

[40] Herm LV, Janiesch C, Helm A, Imgrund F, Hofmann A, Winkelmann A (2023). A framework for implementing robotic process automation projects. Inf. Syst. e-Bus. Manag..

[41] Huang F, Vasarhelyi MA (2019). Applying robotic process automation (RPA) in auditing: A framework. Int. J. Acc. Inf. Syst..

[42] Brown C (2019). Why and how to employ the SIPOC model. J. Bus. Contin. Emerg. Plan..

[43] Coskun, A. (Ed.). Six sigma: Projects and personal experiences. *BoD-Books Demand*10.5772/679 (2011).

[44] Langstrand, J. *An Introduction to Value Stream Mapping and Analysis*. https://reurl.cc/YE9qKn (2016).

[45] George, M. L., & George, M. *Lean Six Sigma for Service*. https://reurl.cc/XG4RK7 (McGraw-Hill, 2003).

